# Fluid Abilities and Rule Learning: Patterning and Biconditional Discriminations

**DOI:** 10.3390/jintelligence6010007

**Published:** 2018-02-27

**Authors:** Irina Baetu, Nicholas R. Burns, Elsa Yu, A. G. Baker

**Affiliations:** 1 School of Psychology, University of Adelaide, Adelaide, SA 5005, Australia; nicholas.burns@adelaide.edu.au; 2 Department of Psychology, McGill University, Montréal, QC H3A 1G1, Canada; yhaoyou@gmail.com (E.Y.); andy.baker@mcgill.ca (A.G.B.)

**Keywords:** associative learning, fluid abilities, positive and negative patterning, biconditional discrimination, configural processing, rule-based generalization

## Abstract

Previous experience with discrimination problems that can only be solved by learning about stimulus configurations enhances performance on new configural discriminations. Some of these effects can be explained by a shift toward increased configural processing (learning about combinations of cues rather than about individual elements), or by a tendency to generalize a learned rule to a new training set. We investigated whether fluid abilities influence the extent that previous experience with configural discriminations improves performance on subsequent discriminations. In Experiments 1 and 2 we used patterning discriminations that could be solved by applying a simple rule, whereas in Experiment 3 we used biconditional discriminations that could not be solved using a rule. Fluid abilities predicted the improvement on the second training set in all experiments, including Experiment 3 in which rule-based generalization could not explain the improvement on the second discrimination. This supports the idea that fluid abilities contribute to performance by inducing a shift toward configural processing rather than rule-based generalization. However, fluid abilities also predicted performance on a rule-based transfer test in Experiment 2. Taken together, these results suggest that fluid abilities contribute to both a flexible shift toward configural processing and to rule-based generalization.

## 1. Introduction

Generalization based on previous learning experiences is an important aspect of intelligence that can, in principle, generate adaptive behaviours. The ability to learn from previous experiences and apply this knowledge to new experiences could rely on a reasoning system that performs computations on mental representations of the perceived events to generate a logical conclusion or judgment [1]. In particular, previous experiences could result in learning the laws, or rules, that seem to generate the perceived events. Such rule learning could be particularly useful for predicting future events that might follow the same pattern. Alternatively, generalization could rely on an associative system that is generally thought to be automatic and less computationally demanding. From this perspective, generalization can be described as resulting from the partial activation of previously learned associations when the current stimuli share some similarities (or features) with stimuli that were experienced in the past (e.g., [2]).

Students of associative learning regularly study rule-based generalization using patterning discriminations [3]. In a positive patterning discrimination, participants learn by trial and error that a combination of two cues, say A and B, is always followed by an outcome (AB+) but when presented separately neither is followed by an outcome (A− and B−). On the other hand, in a negative patterning discrimination the individual cues are followed by the outcome (C+ and D+) but their combination is not (CD−). 

Although some associative accounts can explain this type of learning (e.g., [4,5]), positive and negative patterning can, in principle, be solved by applying the ‘opposites’ rule whereby the outcome on compound trials is the opposite to the outcome that follows the individual elements [6]. If participants learn this rule, then it is possible that they transfer or generalize it to other cues. For example, having experienced a positive patterning discrimination (A−, B−, AB+) and X− and Y− trials, they might predict that the outcome should occur on XY trials even though they have never seen that combination before. Alternatively, when presented with a novel combination of cues, individuals might generalize on the basis of their experience with the individual features of the XY compound, in which case they might expect similar or even stronger outcomes on compound and single element trials (e.g., they might expect no outcome to occur on a XY trial after experience with X− and Y− trials).

People’s performance on these transfer tests is *prima facie* consistent with rule-based generalization (e.g., [3]) but this type of generalization seems to require more cognitive resources than feature-based generalization. This hypothesis is supported by studies showing that rule-based generalization is more likely to occur when there are no time constraints [7] and when learning occurs under low cognitive load [8]. Furthermore, individual differences in fluid abilities have also been shown to correlate with generalization patterns. Wills et al. [9] found that individuals with higher working memory capacity had a greater tendency for rule-based generalization. Maes et al. [10] found a marginally significant positive correlation between rule-based generalization and abstract reasoning ability, although this was not statistically significant when performance on the training set was controlled for. Finally, Don et al. [11] found a positive relationship between rule-based generalization and scores on the Cognitive Reflection Test, which estimates the degree to which individuals’ performance relies on deliberative processes.

Although these studies suggest that individual differences in fluid abilities might influence the extent to which individuals engage in rule-based generalization, the evidence for it is relatively sparse and several questions remain. First, it is still unclear which fluid abilities predict rule-based generalization in patterning discriminations. Previous studies [9,10] that have investigated this issue each assessed only one cognitive ability (working memory or reasoning ability) in relatively small samples (60 or fewer participants). But other fluid abilities, such as processing speed and visuospatial ability, might also influence generalization strategies. In the following experiments, we investigated the relationship between generalization strategies and four fluid abilities: reasoning ability, visuospatial ability, working memory and processing speed.

Second, it is unclear whether fluid abilities influence performance on transfer tests when participants are exposed to novel cue combinations, or whether they also play a role when participants are exposed to new discriminations. Previous studies assessed generalization strategies on transfer tests (e.g., participants were asked whether the outcome would follow the compound XY after experience with X− and Y− trials) but fluid abilities might also determine generalization strategies when participants have the opportunity to learn new patterning discriminations. That is, fluid abilities might influence the extent to which a previously learned discrimination influences learning of a discrimination involving new cues. For example, after having been exposed to positive (A−, B−, AB+) and negative (C+, D+, CD−) patterning discriminations, fluid abilities might influence the acquisition of new patterning discriminations (E−, F−, EF+ and G+, H+, GH−). If so, it would mean that fluid abilities not only influence generalization strategies in ambiguous situations (e.g., on a transfer test in which participants are asked to predict the outcome to a combination of cues they have never experienced) but also in unambiguous situations in which participants receive feedback. Indeed, it is known that learning to solve one discrimination influences performance on a subsequent discrimination [12,13,14]. Our aim was to investigate the role of fluid abilities on the extent to which learning one discrimination influences the acquisition of new discriminations. 

Third, we were interested in whether fluid abilities influence the subsequent acquisition of a discrimination by determining the extent to which participants use a rule learned while solving the first discrimination. Alternatively, it is possible that fluid abilities influence other mechanisms that affect performance, such as increasing the tendency to learn about stimulus configurations (configural processing) rather than about the individual elements of the configuration (elemental processing). Configural processing refers to the idea that combinations of stimuli are perceived as configurations rather than as a combination of the individual elements (e.g., the configuration AB is different from the sum of its individual elements, A and B). These configurations can become associated to outcomes and their association could be different from the associations between the individual elements and the outcome. Interestingly associative models that rely on configural processing (e.g., [5]) can explain positive and negative patterning without assuming that individuals learn rules: the single elements and the compound configuration acquire opposite associations. This might explain why individuals are able to solve these discriminations. It has been suggested that configural and elemental processing lie on a continuum and that individuals can flexibly change their processing style depending on their experience [12,15,16]. That is, it is possible that experience with discriminations that can be solved elementally increases elemental processing, whereas discriminations that can only be solved configurally increase configural processing. This can explain why solving a discrimination that has only a configural solution (i.e., cannot be solved on the basis of the individual cues alone) facilitates the acquisition of subsequent patterning discriminations [12,13,14]. To investigate whether fluid abilities influence performance on a second discrimination by inducing a shift towards configural processing or by increasing the likelihood that a learned rule will be applied to the new discrimination, we tested whether fluid abilities could predict an improvement in performance in situations in which a rule could be learned and applied to the second discrimination (Experiments 1 and 2). We contrasted this with a situation in which this would not be possible and participants would have to rely on configural processing to solve both discriminations (Experiment 3).

In Experiment 1 we tested whether solving patterning discriminations on one set of cues (A−, B−, AB+ and C+, D+, CD−) could subsequently help solve patterning discriminations on a different set of cues (E−, F−, EF+ and G+, H+, GH−). That is, we asked whether learning one set of discriminations could facilitate learning a new set of discriminations. If participants transfer the opposites rule to the new discriminations, then the second discrimination set should be acquired more rapidly. We measured the extent to which performance benefits from this rule generalization by comparing the relative improvement on the second discrimination set to the first. We further tested whether reasoning ability, visuospatial ability, working memory or processing speed predicted improvement in performance of rule-based generalization.

In Experiment 2 we replicated Experiment 1. In addition, we included a set of transfer trials in which some individual cues were followed by feedback (e.g., I−, J−) but the compounds were not (e.g., IJ?). Participants were required to guess the outcome on every trial regardless of whether they received feedback. If they transferred the opposites rule from the fully trained set to the transfer set, then they should make opposite outcome predictions on compound trials (i.e., IJ+). This transfer test is similar to that used in previous studies [3,9,10]. We could therefore also investigate which fluid abilities predict performance on transfer trials. On the basis of previous studies [9,10], we expected at least reasoning ability and working memory to predict performance on the transfer test.

Finally, in Experiment 3 we tested whether the performance advantage on the discriminations trained second was specifically due to rule learning, or whether such an advantage would also pertain when there was no obvious rule that could be learned. In this experiment participants were first trained on a biconditional discrimination (AB+, CD+, AD−, CB−; [17]) before being exposed to a second, similar, discrimination (IJ+, KL+, IL−, KJ−). Unlike positive and negative patterning discriminations that can be solved by applying a simple opposites rule, biconditional discriminations have no simple solution: they cannot be solved on the basis of the individual elements, nor can they be solved by applying a simple rule like the opposites rule. Rather, participants must learn the outcome associated with each configuration of cues. If the improvement in performance on the patterning discriminations trained second in Experiments 1 and 2 was due, at least in part, to factors other than rule learning, such as an increase in configural processing, then the second biconditional discrimination in Experiment 3 should be learned more rapidly than the first despite the fact that participants could not learn an easy rule that would help them solve the second discrimination. Furthermore, if fluid abilities predict an increased shift towards configural processing, then we expected a relationship between fluid abilities and performance improvements on the second biconditional discrimination similar to those found with the patterning discriminations in Experiments 1 and 2. Alternatively, if fluid abilities contribute to an improvement in performance only by facilitating the generalization of a specific rule from one set of discriminations to another, then we expected weaker or absent relationships between fluid abilities and performance improvements on the second biconditional discrimination.

Overall, these experiments allowed us to investigate the relationship between fluid abilities and performance improvements that could be explained by rule-based generalization or an increase in configural processing (in Experiments 1 and 2) and improvements that are not easily accounted for by rule-based generalization but may be explained by an increase in configural processing (Experiment 3). Furthermore, we investigated whether fluid abilities influence generalization strategies in unambiguous situations in which participants are required to learn new discriminations (Experiments 1–3), as well as in more ambiguous transfer tests in which participants do not receive feedback (Experiment 2).

## 2. Materials and Methods

The experiments were approved by the McGill University Human Research Ethics Committee. The computerized tests were coded using Real Studio (Real Software, Austin, TX, USA) and run on 21-inch Apple iMac computers.

### 2.1. Participants

Participants (*N* = 100 in Experiment 1, *N* = 74 in Experiment 2, *N* = 73 in Experiment 3) were students enrolled at McGill University and participated for course credit.

### 2.2. Fluid Abilities Measures

We assessed four fluid abilities: reasoning ability, visuospatial ability, working memory and processing speed. Each fluid ability was estimated from scores on two tests, except for working memory and processing speed, which were each estimated using one test in Experiment 1. The scores on all tests were transformed into z-scores and when two tests were used to assess an ability, an overall ability score was computed as the average of the z-scores. 

#### 2.2.1. Reasoning Ability

Reasoning ability was assessed via two paper-and-pencil tests: an abbreviated version of the Raven’s Advanced Progressive Matrices [18] and the Comprehensive Abilities Battery-Induction (CAB-I), a test of inductive reasoning [19]. The Raven’s matrices test consisted of 12 items. For each item, participants were required to identify the missing element that completes a pattern. Participants were given 15 min to complete the test. Scores consisted of the number of correct items. 

The CAB-I consisted of 12 items, each of which was five groups of four letters. Participants were asked to identify which group of letters did not follow the rule that the other groups of letters followed. Participants were given six minutes to complete the test and the number of correct responses was recorded.

#### 2.2.2. Visuospatial Ability

Visuospatial ability was assessed via two paper-and-pencil tests, the Mental Rotation Test [20] and Paper Folding [21]. Each item of the Mental Rotation Test illustrated a target 3D shape and another four similar shapes. Participants were required to select the two shapes, from the choice of four, that were images of the target shape viewed from different angles. Correct selection of both answers was scored two points, a selection of only one correct answer was scored one point and any other possible answer (e.g., selecting one correct and one incorrect answer) was scored zero points. There were 20 items to complete in 10 min. One participant in Experiment 1 did not complete the test.

The Paper Folding test consisted of 30 items. Each item showed a target pattern and four 3D shapes. Participants identified which of the four 3D shapes could be made from the target pattern. Participants had 12 min to complete the test and the number of correct responses was recorded.

#### 2.2.3. Working Memory

Working memory was assessed using two computerized tests, the Dot Matrix, also known as Spatial Verification Span [22] and the Sentence Span [23]. Only the Dot Matrix was administered in Experiment 1, whereas both tests were administered in Experiments 2 and 3. The Dot Matrix required participants to remember the location of a dot presented on a 5 × 5 grid while verifying a matrix equation (one equation-grid pair). There were four levels, with participants required to perform between two and five equation-grid pairs before indicating the location of those dots on a blank grid. The number of correct location selections was recorded.

The Sentence Span consisted of a series of trials on which participants saw an alternating sequence of sentences and to-be-remembered consonants. The participants had to judge whether each sentence was true or false within four seconds, while attempting to remember the consonants for later recall. There were between four and eight consonant-sentence pairs on each trial, followed by a recall test that asked participants to type the sequence of consonants in the correct order. There were three trials per level for a total of 15 trials. The proportion of correctly remembered letters was recorded for each trial and the total score on the task was the average proportion correct in all trials.

#### 2.2.4. Processing Speed

Processing speed was estimated from two paper-and-pencil tests, the Digit Symbol test from the Wechsler Adult Intelligence Scales [24] and the Visual Matching test from the Woodcock-Johnson Psycho-Educational Battery-Revised [25]. Only the Digit Symbol test was administered in Experiment 1, whereas both tests were administered in Experiments 2 and 3. The Digit Symbol test required participants to fill in blank cells according to a key. Each blank cell was displayed below a digit and participants had to draw the symbol corresponding to the digit. There were 90 items and participants completed as many items as possible in two minutes. Two participants in Experiment 1 and four participants in Experiment 3 did not complete the Digit Symbol test.

The Visual Matching test required participants to search for pairs of numbers. Each item consisted of six numbers, two of which were identical and had to be circled. There were 60 items and participants completed as many items as possible in three minutes.

### 2.3. Learning Tasks

**Figure 1:**
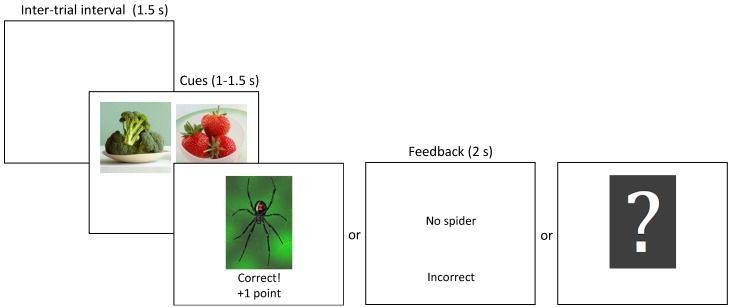
Trial procedure. After a 1.5-s inter-trial interval one or two food cues appeared for a fixed duration of 1 s (Experiments 1 and 2) or 1.5 s (Experiment 3). During this time participants were required to press ‘a’ or ‘l’ on the keyboard to indicate whether they thought the spider picture would follow or not. Regardless of whether they made a response, a feedback screen was shown for 2 s. On trials with feedback, the feedback screen consisted of either the picture of the spider or the words ‘No spider,’ along with the words ‘Correct! +1 point’ or ‘Incorrect.’ On trials without feedback a question mark was presented instead. On all trial types, if no response was made during cue presentation, the words ‘Press A or L faster!’ were displayed.

Participants in each experiment completed a computerized learning task that assessed their ability to learn to discriminate between cues that were followed by an outcome and cues that were not. The cues consisted of pictures of foods and the outcome was the picture of a spider. On every trial participants were shown one or two food cues for 1 s in Experiments 1 and 2, or 1.5 s in Experiment 3 and were required to press ‘a’ or ‘l’ on the keyboard (allocated randomly for each participant) to indicate whether they thought the spider picture would follow or not (Figure 1). Regardless of whether they made a response during the cue presentation, a 2-s feedback screen followed. On most trials, the feedback screen consisted of either the picture of the spider or the words ‘No spider,’ along with the words ‘Correct! +1 point’ or ‘Incorrect,’ which indicated whether the previously made response was correct, or not. In Experiment 2 participants were informed that they would not receive feedback on some trials but that they should respond on these trials even if they were guessing. On no-feedback trials a question mark was shown in place of the normal feedback. If participants did not make a valid response during cue presentation, then the message ‘Press A or L faster!’ was displayed on both feedback and no-feedback trials. The trials were separated by a blank screen during the 1.5-s inter-trial interval.

The experimental designs and types of trials and their outcomes for the learning tasks are summarized in Table 1. Experiments 1 and 2 included positive and negative patterning discriminations whereas Experiment 3 included biconditional discriminations. Cue-picture assignment was randomly determined for each participant. Each trial type was shown 10 times in Experiments 1 and 2 and 16 times in Experiment 3. The trials were randomly intermixed and there was no obvious transition between phases.

Because initial pilot testing revealed that the biconditional discriminations were more difficult to acquire than the patterning discriminations, cue duration was increased in Experiment 3 from 1 s to 1.5 s and the number of training blocks per phase was increased from 10 to 16. The results of the three experiments subsequently confirmed that the biconditional discriminations were more difficult even after the adjustments mentioned above.

Performance on the training sets was quantified as a discrimination score. We first computed a discrimination score for each discrimination as the difference between the proportion of spider predictions for trials that were followed by the spider picture and the proportion of spider predictions for trials that were not followed by the spider picture. For example, in Phase 1 the positive patterning discrimination score was computed as the difference between the proportion of correct spider predictions on AB trials which were paired with the spider [p(spider|AB)] minus the mean proportion of incorrect spider predictions on A and B trials {[p(spider|A) + p(spider|B)]/2}. Thus, the discrimination score for positive patterning was:

Discrimination Score Positive Patterning = p(spider|AB) − [p(spider|A) + p(spider|B)]/2

For the negative patterning discrimination (CD− C+ D+) the individual features were correct, so the difference between elements and compound was reversed:

Discrimination Score Negative Patterning = [p(spider|C) + p(spider|D)]/2 − p(spider|CD)

The discrimination scores for Experiments 1 and 2 for the positive and negative patterning discriminations were averaged. The discrimination scores for Experiment 3 were computed similarly. For example, in Phase 1 (AB+ CD+ AD− CB−) the discrimination score was computed as the difference between the average proportion of correct spider predictions on AB and CD trials minus the average proportion of incorrect spider predictions on AD and CB trials:

Discrimination Score = [p(spider|AB) + p(spider|CD)]/2 − [p(spider|AD) + p(spider|CB)]/2

This resulted in two discrimination scores for each experiment, one for the discriminations trained in Phase 1 and one for the discriminations trained second (in Phase 2 or 3). We could therefore analyse the performance improvement on the discriminations trained second relative to the discriminations trained first by comparing these two scores.

In Experiment 2 we also computed a transfer score for each phase using the predictions made on trials that were not followed by feedback. Following Maes et al. [10], we defined the transfer score as the proportion of outcome predictions consistent with rule-based generalization. For example, the transfer scores for Phase 1 were computed as the proportion of spider predictions on IJ trials and no-spider predictions on KL trials:

Transfer Score = [p(spider|IJ) + p(no spider|KL)]/2

Transfer scores above 0.5 indicated a stronger tendency for rule-based generalization than feature-based generalization, whereas transfer scores below 0.5 indicated a stronger tendency for feature-based generalization.

It is worth noting that performance on transfer tests is influenced by the extent to which the original discriminations are learned [3,10,26]. Some previous studies have attempted to control for individual differences in the speed of acquisition of the first discrimination by implementing a performance criterion whereby participants do not proceed to the transfer test unless they have reached a minimum performance criterion (e.g., [10]). Such a procedure, however, introduces additional confounds, such as increased familiarity with the cues which could change the way they are processed [26,27], or fatigue, in those participants who require more trials to solve the discrimination. We therefore gave all participants the same amount of training. But to control for potential differences in the extent to which the fully trained discriminations were learned, we regressed the transfer scores on fluid abilities, as well as performance on the training sets, age and gender to control for their potential confounding effects. Similarly, we regressed the improvement on the second trained set described above on fluid abilities, as well as performance on the first discrimination set, age and gender.

## 3. Results

### 3.1. Improvement on the Second Training Set

**Figure 2:**
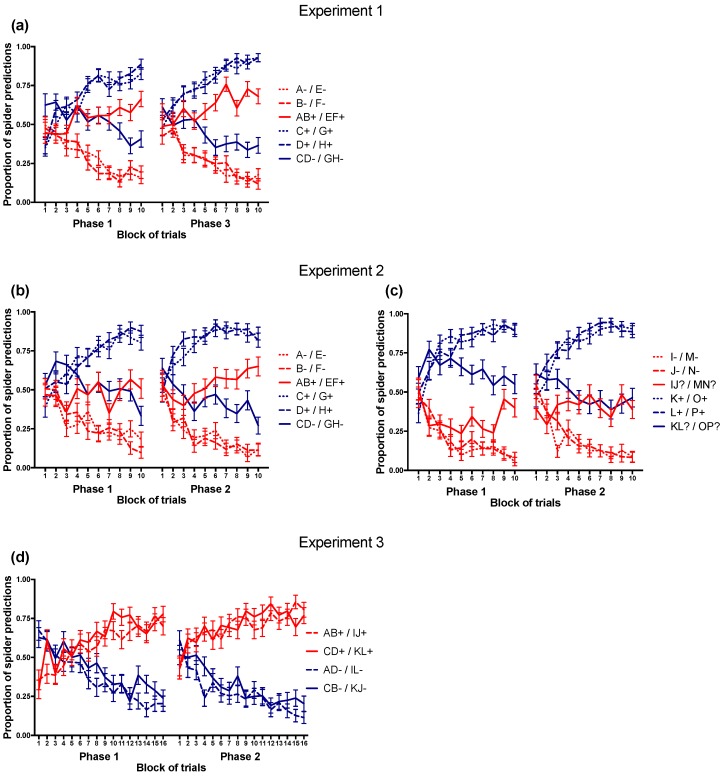
Average trial-by-trial predictions in the learning tasks. Spider predictions were coded as 1 and no-spider predictions were coded as 0. Error bars represent the standard error of the mean. The left panels show the performance on trials from the training sets in (**a**) Experiment 1, (**b**) Experiment 2 and (**d**) Experiment 3. The right panel (**c**) shows the performance on trials from the transfer set in Experiment 2.

Table 2 summarizes the demographic and behavioural data and Figure 2 shows the discrimination performance in each experiment. The discrimination scores on the sets trained second increased relative to the Phase 1 discrimination scores in all three experiments; *t*(99) = 4.47, *p* < 0.001, CI_95_ [0.067 0.173]; *t*(73) = 6.18, *p* < 0.001, CI_95_ [0.107 0.209]; and *t*(72) = 5.27, *p* < 0.001, CI_95_ [0.116 0.257] for Experiments 1, 2 and 3, respectively.

**Figure 3:**
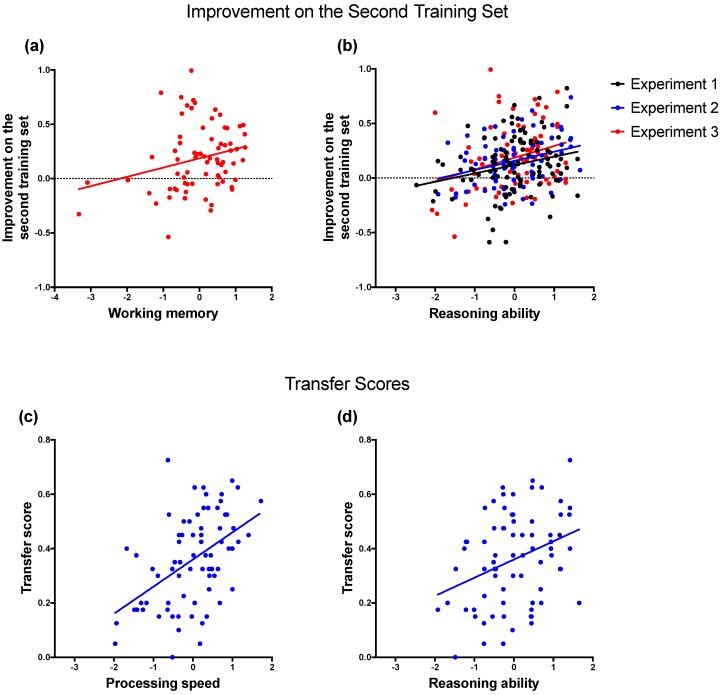
Scatter plots showing the correlations between learning measures and fluid abilities that were significant predictors after controlling for at least age and gender (Table 4). The upper panels illustrate the relationship between the improvement on the second training sets and (**a**) working memory in Experiment 3 and (**b**) reasoning ability in Experiment 1–3. The lower panels illustrate the relationship between the transfer scores in Experiment 2 and (**c**) processing speed and (**d**) reasoning ability.

Table 3 lists the correlations between the learning measures and fluid abilities. We estimated a series of multiple regression models in which the performance improvement on the second training set was regressed on each fluid ability, as well as the potentially confounding factors age, gender and learning performance on the Phase 1 training set. Including the performance on the Phase 1 training set as a predictor ensured that our results were not confounded by the extent to which the original discriminations were learned, which has been shown to influence rule-based generalization (e.g., [3,10]). Note, however, that excluding this predictor from the regression models did not change the pattern of results reported below (compare Table 4 and Table 5). Reasoning ability predicted the improvement in performance on the patterning discriminations (Experiments 1 and 2) and the biconditional discrimination (Experiment 3; see Figure 3b). Working memory was also a predictor of the performance improvement on the second biconditional discrimination (Experiment 3; Figure 3a) but not on the patterning discriminations (Experiments 1 and 2).

**Figure 4:**
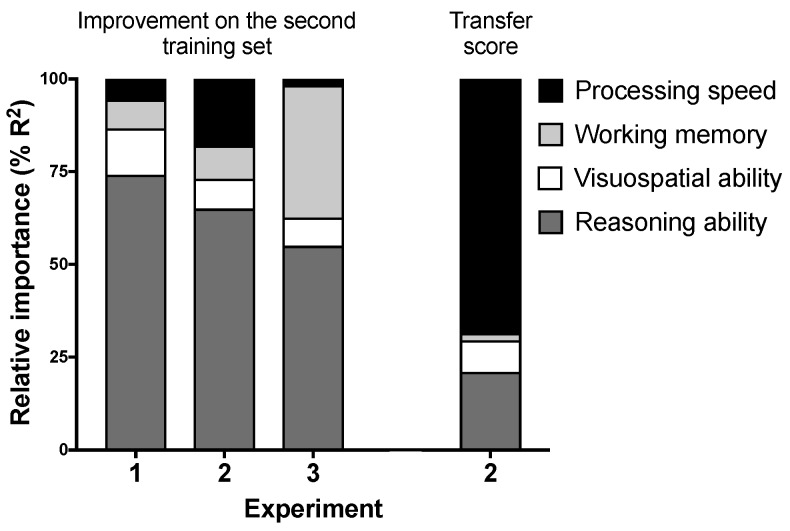
Relative importance metrics for the four fluid abilities obtained from relative importance regression models that included the four fluid abilities as predictors of either the improvement on the second training set (first three bars) or the transfer scores in Experiment 2 (fourth bar).

We also ran relative importance regression models that regressed the improvement on the second training set in each experiment on all four fluid abilities simultaneously (excluding the confounding variables). This allowed us to determine the relative contribution of each fluid ability to the overall R^2^ in a multiple regression model [28]. The first three bars in Figure 4 show the relative contribution of the four fluid abilities in explaining the improvement scores. All models were significant or were close to the significance level, R^2^ = 0.10, *F*(4, 93) = 2.50, *p* = 0.048; R^2^ = 0.12, *F*(4, 69) = 2.41, *p* = 0.057; and R^2^ = 0.14, *F*(4, 68) = 2.85, *p* = 0.030 for Experiments 1, 2 and 3, respectively. Reasoning ability accounted for over half of the explained variability in improvement scores in all experiments (55–74% of R^2^). Working memory accounted for 36% of the total R^2^ in Experiment 3. The other abilities had modest contributions (<18% explained variability).

**Figure 5:**
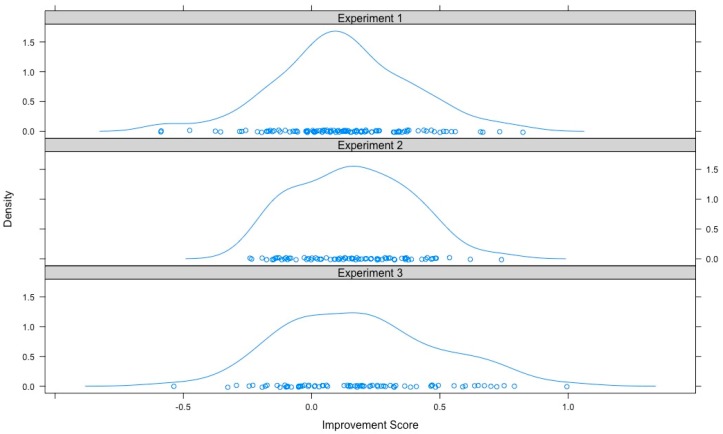
Density plots for improvement on the second training set for Experiments 1, 2 and 3. Pairwise Kolmogorov-Smirnov tests showed that the distributions were not statistically different from each other (minimum *p* = 0.41) and one-way ANOVA showed that the means of the improvement scores were not statistically different from each other, *F*(2, 244) = 1.37, *p* = 0.26.

Finally, we combined the data from the three experiments, as follows. First, we established that improvements on the second training sets were comparable across experiments via one-way ANOVA with improvement as the dependent variable and experiment as the independent variable. Mean improvements on the second training sets were not significantly different across experiments, *F*(2, 244) = 1.37, *p* = 0.26. We also compared the distributions of improvement scores in pairwise fashion via Kolmogorov-Smirnov tests and found no significant differences (minimum *p* = 0.41; see Figure 5). Second, we estimated latent general fluid ability (*g*F) using all available measures across the three experiments in MPlus v7.3 [29]. The fit of the measurement model was good, *χ*^2^(18) = 23.5, *p* = 0.17, RMSEA = 0.04 and CFI = 0.98. Third, we estimated two models: in the first we regressed *g*F on age and gender and performance improvement on *g*F; in the second model, we additionally also regressed *g*F on performance on the Phase 1 training set. In both these models, the regression of performance improvement on the second training set on *g*F was statistically significant (regression coefficient = 0.050, CI_95_ [0.009 0.091], *SE* = 0.021, *p* = 0.017 for the model controlling for age and gender; and regression coefficient = 0.047, CI_95_ [0.017 0.077], *SE* = 0.015, *p* = 0.002 for the model controlling for age, gender and performance on the Phase 1 training set). Thus, a general factor of fluid intelligence predicted the performance improvement on the discriminations trained second, even when performance on the Phase 1 discriminations was controlled for.

### 3.2. Transfer Scores in Experiment 2

The transfer scores in Experiment 2 were significantly smaller than 0.5 in both Phase 1 and Phase 2, *t*(73) = 9.19, *p* < 0.001, CI_95_ [0.260 0.345] and *t*(73) = 3.36, *p* = 0.001, CI_95_ [0.368 0.466] for Phases 1 and 2, respectively. This indicates that on average participants were more likely to demonstrate feature-based generalization rather than rule-based generalization, although the transfer scores changed in the direction of more rule-based generalization from Phase 1 to Phase 2, *t*(73) = 4.54, *p* < 0.001, CI_95_ [0.064 0.164].

We further analysed whether the overall performance on the transfer sets in Experiment 2 could be predicted by fluid abilities. The average transfer scores were regressed on each fluid ability along with age and gender (Table 4). A second set of regression analyses also included the average performance on the training sets in the two phases as an additional predictor to control for its potential confounding influence (Table 5). Processing speed predicted an increase in the transfer scores consistent with rule-based generalization (Figure 3c). Reasoning ability did not significantly predict the transfer scores when performance on the training sets was included in the model (Table 5) but it did have a significant effect when performance on the training sets was excluded from model (Table 4; Figure 3d).

We also ran a relative importance regression model that regressed the transfer scores on the four fluid abilities (see the last bar in Figure 4). The model significantly predicted transfer performance, R^2^ = 0.27, *F*(4, 69) = 6.22, *p* < 0.001. Processing speed and reasoning ability accounted for 69% and 21% of the total R^2^, respectively.

## 4. Discussion

### 4.1. Improvement on the Second Training Set

Similar to previous studies [12,13,26,30], our results suggest that configural processing is flexible and increases after exposure to configural discriminations. This flexibility is thought to be advantageous, as it allows individuals to adapt their processing style depending on which strategy is more useful for learning contingencies between stimuli in the environment. We extend previous findings by demonstrating that individual differences in fluid abilities predict the extent to which an individual will benefit from previous exposure to configural discriminations, when performance on the original discriminations is controlled for. Because reasoning ability predicted this performance increase in Experiments 1 and 2, in which participants could have learned and generalized an opposites rule, and in Experiment 3, in which they could not use a simple rule to solve the discriminations, we propose that reasoning ability is more likely to influence the shift to configural processing. This explains the results of all three experiments, whereas rule-based generalization only explains the results of Experiments 1 and 2. 

There were, however, some differences between experiments. Whereas reasoning ability predicted the performance improvement in all experiments, working memory only predicted it in Experiment 3. One possible explanation is that the biconditional discriminations were more difficult to solve than the patterning discriminations, thus increasing working memory demands and making it more likely that we would observe a relationship between working memory capacity and performance improvements in Experiment 3. Indeed, comparing the discrimination scores in Experiments 1 and 2 to those in Experiment 3 computed from the first 10 blocks of training (to equate the amount of training in all experiments) revealed that the discrimination scores in Experiment 3 were lower than those in Experiments 1 and 2 in Phase 1, minimum *t*(145) = 3.06, *p* = 0.003, CI_95_ [0.039 0.180]. Phase 2 discrimination scores, however, did not differ between experiments, maximum *t*(145) = 0.843, *p* = 0.401, CI_95_ [−0.054 0.135].

### 4.2. Transfer Scores in Experiment 2

Our findings regarding the performance on the transfer sets in Experiment 2 can be compared to previous studies that used similar transfer tests. First, we found that reasoning ability modestly predicted performance on the transfer sets. This is consistent with the results from Maes et al. [10], who also found performance on the Raven’s Standard Progressive Matrices to modestly predict rule-based generalization following patterning discrimination training but only if performance on the training sets was not controlled for. Second, we found that working memory did not predict performance on the transfer sets. This is inconsistent with Wills et al.’s [9] results, which found that individuals with high working memory capacity showed rule-based generalization, whereas individuals with low working memory capacity showed feature-based generalization. The fact that we did not replicate their finding could be due to the data analysis: whereas we analysed the full range of working memory scores using linear regression, Wills et al. only analysed the upper and lower quartiles of their sample on the working memory task (approximately 10 participants in each group) and consequently omitted the data from the 50% of their participants who fell in the middle range. An analysis similar to Wills et al. on our dataset using extreme quartiles, still did not reveal a significant working memory effect. This was true even if only the Phase 1 transfer scores were analysed, reflecting the fact that the procedure used by Wills et al. consisted of only one training phase.

Instead, we found that processing speed strongly predicted transfer scores that are consistent with rule-based generalization. It is possible that processing speed predicted rule-based generalization in our Experiment 2 because we used a speeded task in which participants only had one second to make a response. This could have placed an additional burden on processing that was not present in previous studies that used self-paced transfer tests, potentially explaining why the transfer scores in Experiment 2 were predicted so well by processing speed.

The speeded response requirement might also explain why the transfer scores were on average below 0.5, which indicates that participants were more likely to respond to the transfer compounds based on feature generalization than on the opposites rule. If feature-based generalization is less cognitively demanding than rule-based generalization, this might explain why we found that, on average, participants engaged in feature-based generalization, whereas other studies that used self-paced tests found a stronger tendency for rule-based generalization [3,7,8,10]; but see [26]. Consistent with the idea that rule-based generalization is more demanding, Cobos et al. [7] found that self-paced ratings on a transfer test reflected rule-based generalization but performance on a cued-response priming test was more consistent with feature-based generalization. The authors argued that feature-based generalization relies on fast associative mechanisms, whereas rule-based generalization relies on slower deliberative processes that require executive control and is thus more likely to occur under untimed conditions. It is possible that the speeded response requirement in our experiments decreased the likelihood that participants could engage in rule-based generalization by preventing these deliberative processes. Furthermore, our results suggest that participants could engage in rule-based generalization if their processing speed was sufficiently high, which would presumably allow more time for rule-based generalization processes.

### 4.3. Rule-Based Generalization vs. Configural Processing

As explained in the introduction, we were interested in investigating whether fluid abilities increase the likelihood that participants will use a rule to solve new discriminations or whether they contribute to a shift to configural processing following experience with discriminations that can be solved by learning about stimulus configurations. Taken together, the results of the three experiments suggest that reasoning ability is likely to predict a stronger shift towards configural processing following experience with configural problems. This is because reasoning ability consistently predicted the improvement on the second training set in all experiments, including Experiment 3 that ruled out the possibility that the improvement was due to rule-based generalization. Furthermore, reasoning ability only modestly predicted transfer scores consistent with rule-based generalization in Experiment 2 and this effect was not significant when performance on the training sets was controlled for. Thus overall, these results suggest that reasoning ability is more likely to generate a shift toward configural processing following experience with configural discriminations than encourage rule-based generalization.

Whereas reasoning ability consistently predicted the improvement on the second training set in all experiments, working memory only did so in Experiment 3, which involved more difficult biconditional discriminations. Although speculative, it is possible that working memory is a less reliable predictor of a shift toward configural processing than reasoning ability and this effect only reached the significance level when participants were exposed to more difficult discriminations.

The relationship between fluid abilities and the transfer scores in Experiment 2, however, cannot be explained by the possibility that fluid abilities only increase configural processing. That is, if fluid abilities increase the likelihood that stimuli will be processed configurally after experiencing configural problems, then one would expect subsequent configural problems to be solved more easily but one would not necessarily expect an increase in rule-like responses to the transfer stimuli that were not followed by feedback. This is because an increase in configural processing would merely help participants learn nonlinear discriminations (so it would improve performance on feedback trials) but it would not help them ‘deduce’ which outcome should occur on transfer trials (so it would not influence performance on no-feedback trials). It is therefore more likely that fluid abilities influenced the extent to which participants would use a rule-based strategy to make predictions on the ambiguous trials without feedback.

We found, once performance on the training sets was controlled for, that only processing speed strongly predicted the transfer scores in Experiment 2. However, processing speed did not have a significant effect on the improvement on the second training set in either experiment. This suggests that processing speed might determine the extent to which an individual is capable of applying a learnt rule in ambiguous situations in which feedback is omitted, so performance cannot rely on de novo learning. Furthermore, if one assumes that such rule-based generalization is computationally demanding [7], then this might explain why this effect was particularly evident in our study because we used a speeded response task that imposed a relatively heavy burden on processing speed. So, the difficulty in generating rule-based generalization might have stemmed from the requirement to inhibit a more automatic feature-based generalization tendency rather than from discovering the simple opposites rule. Thus, rule-based generalization in our task might have depended more heavily on the ability to apply the simple rule within a short timeframe rather than on the ability to learn the rule. Had we used a more complex rule and given participants more time to respond, then reasoning ability rather than processing speed may have predicted rule-based generalization.

Nevertheless, although different procedures likely influence the extent to which specific fluid abilities contribute to performance, our results demonstrate that fluid abilities predict the extent to which an individual will experience a shift toward configural processing following training with configural discriminations. They also predict a stronger tendency to exhibit rule-based rather than feature-based generalization on transfer tests. Future research might further investigate the conditions in which specific fluid abilities play a role in experience-induced shifts in processing strategies or rule-based generalization.

## Figures and Tables

**Table 1:** Learning tasks designs ^1^.

	Phase 1	Phase 2	Phase 3
Experiment 1			
Training set:			
Positive patterning	A−, B−, AB+		E−, F−, EF+
Negative patterning	C+, D+, CD−		G+, H+, GH−
Other trials	I+, O+	IJ+, KL+, MN+	K+, Q+
	U−, V−	OP−, QR−, ST−	U−, V−
Experiment 2			
Training set:			
Positive patterning	A−, B−, AB+	E−, F−, EF+	
Negative patterning	C+, D+, CD−	G+, H+, GH−	
Transfer set:			
Positive patterning	I−, J−, IJ?	M-, N-, MN?	
Negative patterning	K+, L+, KL?	O+, P+, OP?	
Experiment 3			
Training set:			
Biconditional discrimination	AB+, CD+, AD−, CB−	IJ+, KL+, IL−, KJ−	
Other trials	EF+, GH−	MN+, OP−	

^1^ There were 10 trials of each type in Experiments 1 and 2 and 16 trials of each type in Experiment 3. “+” indicates that the spider picture followed the cue(s), “−” indicates that the spider picture did not follow the cue(s) and “?” indicates that no feedback was given.

**Table 2:** Demographic and Behavioural Data ^1^.

	Experiment 1 ^2^	Experiment 2	Experiment 3
Demographic Data			
Age	20.89 (1.48)	21.30 (2.46)	20.26 (1.40)
Gender F:M	64:36	54:20	62:11
Fluid Abilities			
Reasoning Ability:			
Raven’s Matrices	8.18 (2.38)	8.24 (2.14)	8.97 (2.39)
CAB-I	9.10 (1.85)	8.85 (2.03)	9.67 (1.77)
Visuospatial Ability:			
Mental Rotation Test	21.59 (7.77)	15.09 (8.57)	21.07 (6.57)
Paper Folding	19.67 (5.09)	19.30 (5.41)	21.22 (4.36)
Working Memory:			
Dot Matrix	36.86 (9.48)	37.55 (9.18)	39.86 (9.78)
Sentence Span		0.69 (0.16)	0.73 (0.17)
Processing Speed:			
Digit Symbol	64.23 (14.83)	67.99 (11.01)	70.14 (11.51)
Visual Matching		54.89 (4.67)	55.81 (5.10)
Learning Performance ^3^			
Training Set Discrimination Scores:			
Phase 1	0.21 (0.23)	0.18 (0.21)	0.22 (0.22)
Phase 2 (or 3)	0.33 (0.23)	0.34 (0.26)	0.40 (0.31)
Improvement	0.12 (0.27)	0.16 (0.22)	0.19 (0.30)
Transfer Scores:			
Phase 1		0.30 (0.18)	
Phase 2		0.42 (0.21)	
Improvement		0.11 (0.22)	

^1^ Standard deviations are indicated in parentheses. ^2^ Only one test of working memory and one test of processing speed were administered in Experiment 1. ^3^ The calculations used to obtain the discrimination and transfer scores are explained in Section 2.3.

**Table 3:** Correlations Between Learning Measures and Fluid Abilities ^1^.

	Improvement on the Second Training Set			Transfer Score
	Experiment 1	Experiment 2	Experiment 3	Experiment 2
Reasoning Ability:	0.24 *	0.32 *	0.29 *	0.33 *
Raven’s Matrices	0.25 *	0.23 *	0.14	0.13
CAB-I	0.16	0.30 *	0.34 *	0.42 **
Visuospatial Ability:	−0.04	0.11	0.01	0.25 *
Mental Rotation Test	−0.06	0.05	0.06	0.15
Paper Folding	−0.02	0.16	−0.04	0.29 *
Working Memory:	−0.01	0.16	0.25 *	0.11
Dot Matrix	−0.01	0.17	0.32 *	0.04
Sentence Span		0.10	0.12	0.15
Processing Speed:	0.09	0.20	0.07	0.48 **
Digit Symbol	0.09	0.15	−0.03	0.48 **
Visual Matching		0.18	0.12	0.30 *

^1^ Reasoning ability, visuospatial ability, working memory and processing speed scores are average z-scores of individual tests. Only one test of working memory and one test of processing speed were administered in Experiment 1. * denotes the correlation is significant at the 0.05 level. ** denotes the correlation is significant at the 0.001 level.

**Table 4:** Results of Linear Models That Regressed the Learning Measures on Each Fluid Ability and Controlled for Age and Gender ^1^.

	Improvement on the Second Training Set			Transfer Score
	Experiment 1	Experiment 2	Experiment 3	Experiment 2
Additional Predictors Included in the Models	Age			Age
	Gender			Gender
Reasoning Ability				
Coefficient	0.085	0.084	0.105	0.057
95% CI	(0.022, 0.148)	(0.020, 0.147)	(0.021, 0.189)	(0.009, 0.105)
SE	0.031	0.032	0.042	0.024
*p*	0.009 *	0.011 *	0.015 *	0.020 *
Visuospatial Ability:				
Coefficient	−0.005	0.020	−0.010	0.034
95% CI	(−0.072, 0.061)	(−0.042, 0.082)	(−0.095, 0.075)	(−0.012, 0.079)
SE	0.034	0.031	0.043	0.023
*p*	0.880	0.516	0.815	0.143
Working Memory:				
Coefficient	0.002	0.037	0.080	0.009
95% CI	(−0.054, 0.059)	(−0.028, 0.101)	(0.002, 0.158)	(−0.040, 0.057)
SE	0.029	0.032	0.039	0.024
*p*	0.934	0.262	0.045 *	0.720
Processing Speed:				
Coefficient	0.030	0.053	0.025	0.099
95% CI	(−0.026, 0.086)	(−0.011, 0.117)	(−0.065, 0.114)	(0.057, 0.142)
SE	0.028	0.032	0.045	0.021
*p*	0.291	0.104	0.587	<0.001 **

^1^ SE = Standard error. * denotes the coefficient is significant at the 0.05 level and ** denotes the coefficient is significant at the 0.001 level.

**Table 5:** Results of Linear Models That Regressed the Learning Measures on Each Fluid Ability and Controlled for Age, Gender and Training Performance ^1^.

	Improvement on the Second Training Set			Transfer Score
	Experiment 1	Experiment 2	Experiment 3	Experiment 2
Additional Predictors Included in the Models	Age			Age
	Gender			Gender
	Phase 1 Training Set Discrimination Score			Average Training Set Discrimination Score
Reasoning Ability				
Coefficient	0.084	0.095	0.120	0.037
95% CI	(0.033, 0.134)	(0.035, 0.155)	(0.042, 0.198)	(−0.010, 0.085)
SE	0.025	0.030	0.039	0.024
*p*	0.001 *	0.002 *	0.003 *	0.121
Visuospatial Ability:				
Coefficient	0.033	0.030	0.015	0.023
95% CI	(−0.022, 0.088)	(−0.029, 0.090)	(−0.066, 0.097)	(−0.021, 0.066)
SE	0.028	0.030	0.041	0.022
*p*	0.232	0.315	0.707	0.305
Working Memory:				
Coefficient	0.011	0.054	0.112	−0.012
95% CI	(−0.035, 0.057)	(−0.008, 0.116)	(0.039, 0.185)	(−0.059, 0.035)
SE	0.023	0.031	0.037	0.023
*p*	*0.626*	*0.090*	0.003 *	*0.612*
Processing Speed:				
Coefficient	0.035	0.060	0.028	0.088
95% CI	(−0.010, 0.080)	(−0.001, 0.122)	(−0.057, 0.113)	(0.046, 0.129)
SE	0.023	0.031	0.043	0.021
*p*	0.126	0.052	0.518	<0.001 **

^1^ SE = Standard error. * Denotes the coefficient is significant at the 0.05 level and ** denotes the coefficient is significant at the 0.001 level.
